# Supplementary figures and images for: Enzyme stoichiometry indicates the variation of microbial nutrient requirements at different soil depths in subtropical forests
Source: PLoS One. 2020 Feb 4;15(2):e0220599. doi: 10.1371/journal.pone.0220599 (PMC6999874; doi:10.1371/journal.pone.0220599)

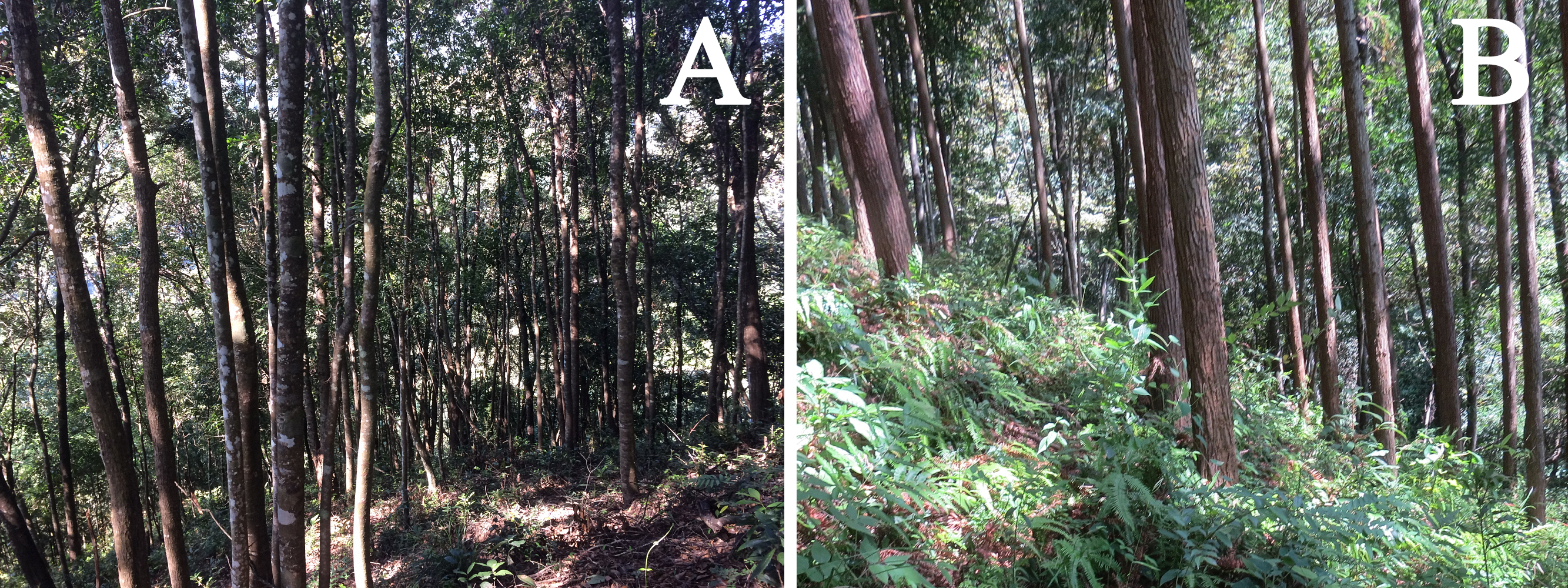

Supplement: S1 Fig — (A) the natural secondary forest (NSF) and (B) the Chinese fir plantation forest (CPF). (TIF) [file pone.0220599.s001.tif]

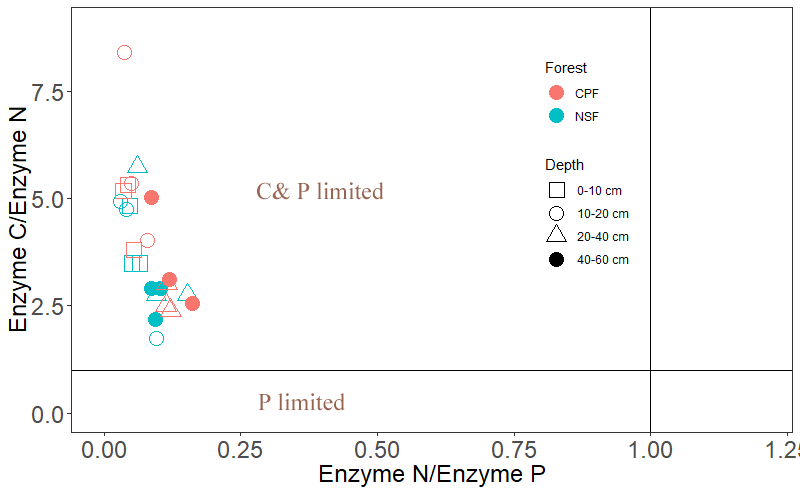

Supplement: S2 Fig — Different symbols represent different soil depth. (PNG) [file pone.0220599.s002.png]
