# Supplementary material for: Enzyme stoichiometry indicates the variation of microbial nutrient requirements at different soil depths in subtropical forests
Source: PLoS One. 2020 Feb 4;15(2):e0220599. doi: 10.1371/journal.pone.0220599 (PMC6999874; doi:10.1371/journal.pone.0220599)
Supplement: S1 Table — Capital letters mean the significant difference between the two forests at the same soil depth, and the different lower cases reflect the significant difference between soil depths within one forest. Values were mean ± standard error (n = 3). BX:β-xylosidase, CBH: β-D-cellobiosidase, BG: β-1,4-glucosidase, NAG: β-1,4-N-acetylglucosaminidase, LAP: L-leucine aminopeptidase. (PDF) [file pone.0220599.s007.pdf]

S1 Table. Individual enzyme activity in the natural secondary forest (NSF) and Chinese fir plantation forest (CPF) at different soil depths

Depth (cm)	BX ($nmol\ g^{-1}$ dry soil h^{-1})		CBH ($nmol\ g^{-1}$ dry soil h^{-1})		BG ($nmol\ g^{-1}$ dry soil h^{-1})		NAG ($nmol\ g^{-1}$ dry soil h^{-1})		LAP ($nmol\ g^{-1}$ dry soil h^{-1})	
	NSF	CPF	NSF	CPF	NSF	CPF	NSF	CPF	NSF	CPF
0-10	**149.9 ±6.1Aa**	**92.4 ±8.4Ba**	**43.9 ±2.9Aa**	**31.8 ±2.2Ba**	360.0 ±53.9Aa	404.8 ±61.5Aa	75.6 ±4.2Aa	55.1 ±4.5Aa	**65.5 ±2.6Aa**	**57.0 ± 3.0Bab**
10-20	74.9 ± 13.5Ab	78.1 ± 19.9Aa	24.3 ±0.9Ab	19.9 ± 2.0Ab	227.7 ±60.0Aab	302.5 ±6.7Aa	59.3 ± 7.3Ab	39.0 ± 4.8Aab	34.0 ±6.6Ab	33.5 ±8.4Ab
20-40	46.1 ±11.3Abc	34.5 ±13.6Ab	15.6 ±2.7Ac	18.7 ± 2.5Ab	277.1 ±60.9Aab	191.5 ± 5.6Ab	36.4 ±2.5Ac	31.9 ±3.8Acdc	56.8 ±3.4Aa	60.6 ± 3.0Aa
40-60	21.9 ± 3.3Ac	14.5 ±4.3Ab	10.8 ±2.0Ac	13.7 ± 1.6Ab	153.0 ±15.0Ab	175.6 ±13.6Ab	18.3 ±1.7Ad	17.7 ± 4.6Ad	52.1 ±8.1Aab	42.8 ±11.7Aab

Capital letters mean the significant difference between the two forests at the same soil depth, and the different lower cases reflect the significant difference between soil depths within one forest. Values were mean ± standard error (n=3). BX：β-xylosidase, CBH： β-D-cellobiosidase, BG： β-1,4-glucosidase, NAG： β-1,4-N-acetylglucosaminidase, LAP： L-leucine aminopeptidase.
