# Supplementary material for: Enzyme stoichiometry indicates the variation of microbial nutrient requirements at different soil depths in subtropical forests
Source: PLoS One. 2020 Feb 4;15(2):e0220599. doi: 10.1371/journal.pone.0220599 (PMC6999874; doi:10.1371/journal.pone.0220599)
Supplement: S2 Table — Note: Moisture: soil moisture, SOC: soil organic carbon, TN: total nitrogen, TP: total phosphorus, BG: β-1,4-glucosidase, CBH: β-D-cellobiosidase, BX: β-xylosidase, NAG: β-1,4-N-acetylglucosaminidase, LAP: L-leucine aminopeptidase, AP: acid phosphatase. Correlations were considered significant (*) at p < 0.05 (two-tailed) and highly significant (**) at p < 0.01 (two-tailed). (PDF) [file pone.0220599.s008.pdf]

S2 Table. **Spearman correlation coefficients (ρ) relating extracellular enzyme activities with soil chemical properties and nutrient stoichiometry**.

Enzyme activity (nmol g soil^{-1} h^{-1})	pH	Moisture (%)	SOC (g kg^{-1})	TN (g kg^{-1})	TP (g kg^{-1})	SOC/TN	SOC/TP	TN/TP
BG+BX+CBH	-0.364	0.682**	0.825**	0.827**	0.482*	0.625**	0.689**	0.580**
LAP+NAG	-0.352	0.559**	0.788**	0.808**	0.267	0.423*	0.694**	0.688**
AP	-0.201	0.695**	0.908**	0.864**	0.417*	0.671**	0.781**	0.656**
BX	-0.241	0.517**	0.917**	0.892**	0.435*	0.667**	0.790**	0.699**
CBH	-0.094	0.543**	0.864**	0.832**	0.470*	0.671**	0.755**	0.646**
BG	-0.380	0.688**	0.712**	0.727**	0.474*	0.556**	0.585**	0.474*
NAG	-0.188	0.541**	0.879**	0.865**	0.336	0.621**	0.781**	0.708**
LAP	-0.411*	0.249	0.135	0.193	-0.004	-0.153	0.114	0.217

Note: Moisture: soil moisture, SOC: soil organic carbon, TN: total nitrogen, TP: total phosphorus, BG: β-1,4-glucosidase, CBH: β-D-cellobiosidase, BX: β-xylosidase, NAG: β-1,4-N-acetylglucosaminidase, LAP: L-leucine aminopeptidase, AP: acid phosphatase. Correlations were considered significant (*) at $p < 0.05$ (two-tailed) and highly significant (**) at $p < 0.01$ (two-tailed).
