# Supplementary material for: Enzyme stoichiometry indicates the variation of microbial nutrient requirements at different soil depths in subtropical forests
Source: PLoS One. 2020 Feb 4;15(2):e0220599. doi: 10.1371/journal.pone.0220599 (PMC6999874; doi:10.1371/journal.pone.0220599)
Supplement: S3 Table — Moisture: soil moisture, SOC: soil organic carbon, TN: soil total nitrogen, TP: soil total phosphorus, BG: β-1,4-glucosidase, CBH: β-D-cellobiosidase, BX: β-xylosidase, NAG: β-1,4-N-acetylglucosaminidase, LAP: L-leucine aminopeptidase, AP: alkaline phosphatase. * Correlation is significant at p < 0.05 (two-tailed); ** Correlation is highly significant at p < 0.01 (two-tailed). (PDF) [file pone.0220599.s009.pdf]

S3 Table. Spearman correlation coefficients (ρ) relating enzyme stoichiometry with soil chemical properties and nutrient stoichiometry.

Enzyme stoichiometry	pH	Moisture (%)	SOC (g kg^{-1})	TN (g kg^{-1})	TP (g kg^{-1})	SOC/TN	SOC/TP	TN/TP
(BG+BX+CBH)/(NAG+LAP)	-0.210	0.505*	0.543**	0.530**	0.512*	0.531**	0.415*	0.281
(BG+BX+CBH)/ AP	-0.172	-0.465*	-0.636**	-0.563**	-0.039	-0.456*	-0.610**	-0.511*
(NAG+LAP)/ AP	0.008	-0.626**	-0.777**	-0.728**	-0.380	-0.641**	-0.668**	-0.515*

Moisture: soil moisture, SOC: soil organic carbon, TN: soil total nitrogen, TP: soil total phosphorus, BG: β-1,4-glucosidase, CBH: β-D-cellobiosidase, BX: β-xylosidase, NAG: β-1,4-N-acetylglucosaminidase, LAP: L-leucine aminopeptidase, AP: alkaline phosphatase. * Correlation is significant at $p < 0.05$ (two-tailed); ** Correlation is highly significant at $p < 0.01$ (two-tailed).
