# Supplementary material for: Enzyme stoichiometry indicates the variation of microbial nutrient requirements at different soil depths in subtropical forests
Source: PLoS One. 2020 Feb 4;15(2):e0220599. doi: 10.1371/journal.pone.0220599 (PMC6999874; doi:10.1371/journal.pone.0220599)
Supplement: S4 Table — Moisture: soil moisture, SOC: soil organic carbon, TN: soil total nitrogen, TP: soil total phosphorus. * Correlation is significant at p < 0.05 (two-tailed); ** Correlation is highly significant at p< 0.01 (two-tailed). (PDF) [file pone.0220599.s010.pdf]

S4 Table. **Spearman correlation coefficients (ρ) relating vector length and angle with soil chemical properties and nutrient stoichiometry**.

Vector characteristics	pH	Moisture (%)	SOC (g kg^{-1})	TN (g kg^{-1})	TP (g kg^{-1})	SOC/TN	SOC/TP	TN/TP
Length	-0.228	0.414*	0.419*	0.425*	0.488*	0.420*	0.301	0.195
Angle	0.131	0.564**	0.754**	0.685**	0.150	0.580**	0.711**	0.583**

Moisture: soil moisture, SOC: soil organic carbon, TN: soil total nitrogen, TP: soil total phosphorus. * Correlation is significant at $p < 0.05$ (two-tailed); ** Correlation is highly significant at $p < 0.01$ (two-tailed).
