# Supplementary material for: Enzyme stoichiometry indicates the variation of microbial nutrient requirements at different soil depths in subtropical forests
Source: PLoS One. 2020 Feb 4;15(2):e0220599. doi: 10.1371/journal.pone.0220599 (PMC6999874; doi:10.1371/journal.pone.0220599)
Supplement: S5 Table — Values represented mean ± standard error (n = 3). Capital letters show the significant difference between the two forests stand at the same soil depth, and the different lower cases reflect the significant difference within one forest stand at four soil depths. (PDF) [file pone.0220599.s011.pdf]

**S5 Table. Vector analysis, using β-1,4-glucosidase (BG) as single C acquiring enzyme, between the natural secondary forest (NSF) and the Chinese fir plantation forest (CPF) at different soil depths**

| Depth | Vector L | | Vector A | |
|---|---|---|---|---|
| (cm) | NSF | CPF | NSF | CPF |
| 0-10 | 0.72±0.03Aa | 0.79±0.03Aa | 80.46±0.57Aab | 80.22±0.65Aa |
| 10-20 | 0.69±0.07Aa | 0.83±0.02Aa | 81.45±0.50Aa | 77.14±1.04Ab |
| 20-40 | 0.77±0.04Aa | 0.70±0.003Ab | 73.90±1.11Ab | 73.92±0.19Ac |
| 40-60 | 0.71 ±0.02Aa | 0.79±0.04Aab | 75.96±0.87Aab | 70.79±0.95Bd |

Values represented mean ± standard error (n=3). Capital letters show the significant difference between the two forests stand at the same soil depth, and the different lower cases reflect the significant difference within one forest stand at four soil depths.
